# Identification and culture of proliferative cells in abnormal *Taenia solium* larvae: Role in the development of racemose neurocysticercosis

**DOI:** 10.1371/journal.pntd.0009303

**Published:** 2021-03-22

**Authors:** Miguel A. Orrego, Manuela R. Verastegui, Carlos M. Vasquez, Uriel Koziol, Juan P. Laclette, Hector H. Garcia, Theodore E. Nash, for the Cysticercosis Working Group in Peru

**Affiliations:** 1 Laboratory of Immunopathology in Neurocysticercosis, Facultad de Ciencias y Filosofía, Universidad Peruana Cayetano Heredia, Lima, Perú; 2 Infectious Diseases Research Laboratory, Facultad de Ciencias y Filosofía, Universidad Peruana Cayetano Heredia, Lima, Perú; 3 Department of Neurosurgery, Instituto Nacional de Ciencias Neurológicas, Lima, Perú; 4 Sección Biología Celular, Facultad de Ciencias, Universidad de la República, Montevideo, Uruguay; 5 Department of Immunology, Institute of Biomedical Research, Universidad Nacional Autónoma de México, Ciudad de México, México; 6 Cysticercosis Unit, Instituto Nacional de Ciencias Neurológicas, Lima, Perú; 7 Laboratory of Parasitic Diseases, National Institute of Allergy and Infectious Diseases, National Institutes of Health, Bethesda, Maryland, United States of America; Universidad de la Republica, Uruguay, URUGUAY

## Abstract

Racemose neurocysticercosis is an aggressive disease caused by the aberrant expansion of the cyst form of *Taenia solium* within the subarachnoid spaces of the human brain and spinal cord resulting in a mass effect and chronic inflammation. Although expansion is likely caused by the proliferation and growth of the parasite bladder wall, there is little direct evidence of the mechanisms that underlie these processes. Since the development and growth of cysts in related cestodes involves totipotential germinative cells, we hypothesized that the expansive growth of the racemose larvae is organized and maintained by germinative cells. Here, we identified proliferative cells expressing the serine/threonine-protein kinase *plk1* by *in situ* hybridization. Proliferative cells were present within the bladder wall of racemose form and absent from the homologous tissue surrounding the vesicular form. Cyst proliferation in the related model species *Taenia crassiceps* (ORF strain) occurs normally by budding from the cyst bladder wall and proliferative cells were concentrated within the growth buds. Cells isolated from bladder wall of racemose larvae were established in primary cell culture and insulin stimulated their proliferation in a dose-dependent manner. These findings indicate that the growth of racemose larvae is likely due to abnormal cell proliferation. The different distribution of proliferative cells in the racemose larvae and their sensitivity to insulin may reflect significant changes at the cellular and molecular levels involved in their tumor-like growth. Parasite cell cultures offer a powerful tool to characterize the nature and formation of the racemose form, understand the developmental biology of *T*. *solium*, and to identify new effective drugs for treatment.

## Author summary

Racemose neurocysticercosis is the most aggressive and lethal form of the disease characterized by progressive expansion of the larval stage of *Taenia solium* within the subarachnoid spaces of the brain. Currently, the cellular or molecular events that promote its formation and development are unknown. Our observations, employing tissue samples, revealed the presence of mitotically active cells in the bladder wall of the racemose larvae. Finally, we isolated and established primary cell cultures and observed that these cells are sensitive (proliferate in response to insulin). These results suggest that the proliferative cells stimulated by host biomolecules like hormones would be responsible for the abnormal growth of racemose larvae. Further studies are needed to identify all genes and pathways altered in the racemose form. The primary cell cultures will be used as a therapeutic target and allow us in future works to understand the developmental biology of the parasite.

## Introduction

Neurocysticercosis (NCC) is the infection of the central nervous system with metacestode larvae (cysticerci or cysts) of the pork tapeworm *Taenia solium* [1]. The life cycle of the parasite is complex and alternates between humans, who harbor the adult tapeworm in their small intestine, and pigs, the obligatory intermediate host [2]. Infective ova are excreted in the feces of the human tapeworm carrier. When free-roaming pigs ingest contaminated feces, the ova hatch; the released embryos reach the bloodstream and characteristically develop into cystic larvae in the muscles, subcutaneous tissues, and the brain [1,2]. The ingestion of undercooked pork allows metacestodes to develop into the adult intestinal tapeworm in humans, completing the life cycle. However, humans may also develop cysticercosis after the accidental ingestion of ova via fecal-oral route [1].

In general, viable cysticerci located in the brain parenchyma invoke little or no inflammatory reaction from the host [3]. However, when the cysts degenerate a capsule made up of infiltrated mononuclear inflammatory cells and connective tissue forms around the cysticercus [4]. This results in deleterious effects on the adjacent regions of the brain, causing astrogliosis, microglial proliferation, edema, neuronal damage, and infiltration of immune cells [3–6]. Increased inflammation around degenerating cysts in the brain parenchyma is frequently associated with seizures [7].

Extraparenchymal infections follow a different course [8]; cysts in the subarachnoid spaces, particularly in the basal cisterns or in the Sylvian fissures, grow continuously into the contiguous spaces to form multivesicular membranous structures (referred to as racemose larvae) with a degenerated or absent scolex [8–11], resulting in mass effects and exacerbated local inflammation [9]. Degenerating but still growing racemose larvae give rise to acute and chronic inflammation leading to infarcts, fibrosis and thickening of the leptomeninges and subsequent nerve entrapments, localized nervous system injury, hydrocephalus, and intracranial hypertension [9,11,12]. Subarachnoid NCC occurs in 15–20% of patients, is more aggressive and difficult to diagnose and treat than the parenchymal single-vesicle form, and is associated with a mortality rate as high as 20% [13,14].

The nature of the racemose larvae is unclear. The incubation is prolonged in the order of 1.5 to 2.5 decades in the usual case, and limited serial observations show expansion of an irregular cystic mass extending from one subarachnoid region into other subarachnoid spaces leading to mass effects and even death [9]. Although hydropic degeneration was suggested as a cause of expansion [15], the histology of the racemose larvae, which commonly consists of characteristic but aberrantly organized [10] cystic vesicular tissue with surface tegument, is not consistent with this hypothesis.

Studies in different species of parasites have evidenced a population of undifferentiated stem cells called germinative cells that are involved in parasite growth and drive the transition between the different life stages of cestodes [16,17]. Additionally, germinative cells are the only mitotically active, self-renewable, and capable of differentiating into any type of somatic cell. The biological functions of germinative cells are poorly understood and their study is complicated by the fact that cestodes have complex life cycles that include more than one host [18].

Studies in *Echinococcus multilocularis* have reported various cell markers that allow identifying germinative cells such as Polo-like Kinase 1 (PLK1) [19], Argonaute 2 (ARGO2), protein Nanos (NANOS) [20], and a novel terminal repeat retrotransposon in miniature (TRIM) [21]. In particular, *plk1* is expressed in late G2 and M phases regulating both mitosis and meiosis.

*Taenia crassiceps* is a related parasite whose adult stage develops in the intestine of canines and its metacestodes in rodents, who acquire the infection after ingesting ova present in feces of the definitive host [22]. The ORF strain proliferates asexually by budding, a large number of cysts can be produced in the peritoneal cavity of mice and therefore are readily available for laboratory investigations [22]. Cells isolated from the proliferative bladder wall were able to regenerate the whole cyst; these findings would suggest the participation of the stem-like cells in the proliferation of the bladder wall during the formation and development of buds for the long-term survival of the parasite within the host [17].

Previously, we identified proliferative cells in the *T*. *solium* vesicular form, these cells were limited to the neck region, which is the expected location of germinative cells since proglottids are formed from this region [23]. Here we show that racemose larvae contain proliferative *plk1*-expressing cells in their bladder wall that drive continuous cyst growth. In contrast, normal vesicular larvae do not have these cells in an analogous location in the bladder wall. Furthermore, we established a viable primary cell culture and show these cells increase proliferation in response to insulin.

## Methods

### Ethics statement

The protocols for the use of animals were reviewed and approved by the Institutional Ethics Committees for the Use of Animals of the Veterinary School of Universidad Nacional Mayor de San Marcos (Protocol number 006) and Universidad Peruana Cayetano Heredia (Protocol numbers 62392 and 101250) in Lima, Peru. Additionally, ethics approval for the use of racemose NCC samples was obtained from de Ethics Committee of Instituto Nacional de Ciencias Neurológicas (INCN) (242-2018-DG-INCN).

### Experimental design

A total of six samples of the racemose larvae and 12 vesicular larvae samples of *T*. *solium* were used to identify in situ germinative cells and observe the distribution of these cells in both forms of cyst. Additionally, portions of racemose larvae were used to isolate and establish a primary culture. Cysts of *T*. *crassiceps* ORF strain were used as controls. A schematic diagram is shown in S1 Fig.

### Sample collection

Portions of racemose larvae removed during medically indicated surgical extraction were recovered from the surgical ward of the INCN in Lima, Peru. The flasks with samples were anonymized (removal of any personal identification of the patient) collected from the operating room and transported to the laboratory in phosphate buffer solution (PBS) pH 7.4.

Samples of vesicular larvae were obtained from naturally infected pigs from an endemic zone of Peru as previously described [24]. The larvae were removed from the skeletal muscle and transported to the laboratory in PBS pH 7.4 (Gibco-Invitrogen, Gaithersburg, MD) supplemented with antibiotics (100 U/ml penicillin and 100 g/ml streptomycin from Gibco-Invitrogen, Gaithersburg, MD)

Cysts of *T*. *crassiceps* ORF strain were provided by Dr. Theodore Nash (Laboratory of Parasitic Diseases, NIAID, NIH, USA) and processed according to Robinson [22]. Six-week-old female BALB/c mice were inoculated intraperitoneally with 10 cysts suspended in RPMI medium. After three months, the animals were euthanized with chloroform and abdominal cysts were collected aseptically for this study.

### Viability

Racemose samples were washed twice with sterile PBS and once with medium (RPMI 1640 with 10 mM HEPES buffer (Gibco), 100 U/ml penicillin, 100 g/ml streptomycin and 0.25 g/ml amphotericin B; all from Gibco-Invitrogen, Gaithersburg, MD) and allowed to stabilize in this medium for 1 h at 37°C in 5% CO2. The medium was removed and samples incubated for 30 min in RPMI 1640 with 500 mM of MitoTracker Red CM-H2Xros (ThermoFisher Scientific, Waltham, MA); then, washed with PBS and stained with a 1% methylene blue solution for 30 seconds. Washed 3 times with PBS to remove the excess unbound dye and observed under a confocal microscope at 599 nm wavelength. Viable portions staining with Mitotracker show a red signal and these were fixed in neutral buffered formalin and paraffin-embedded for histology, used for cell isolation and or preserved in RNAlater (Qiagen) at -70°C

### *In situ* hybridization in tissue

Digoxigenin-labeled LNA type probes (*plk1*; 5’TGTCTGACTCTCGACGTACGAT-3’ and *actin*; 5’-ATCTGCTGGAAGGTGGAGAGT-3’) (Qiagen/Life Technologies, Gaithersburg, MD) were designed and synthesized using the annotated sequence of the *pkl1* (TsM_001148400) and *actin* (TsM_000357600) genes of *T*. *solium* (GeneDB, https://www.genedb.org/#/species/Tsolium) [25]. The *plk1* sense probe was used as a control and included in all experiments. The formalin-fixed paraffin-embedded (FFPE) samples of six racemose larvae were cut in 4 μm sections placed on poly-L-lysine coated slides and thereafter processed [26]. All the solutions used were prepared immediately before use and the water was treated with diethylpyrocarbonate (DEPC) to eliminate any contaminating RNA. Sections were deparaffinized at 56°C, rehydrated in solutions with decreasing proportions of ethanol (100% to 70%), permeabilized by heating in citrate buffer (10 mM citric acid, 0.05% Tween 20, pH 6.0) for 30 min at 95°C, then incubated in 0.2M HCL, post-fixed in 4% PFA in PBS for 20 min, incubated twice for 5 min in 0.1M triethanolamine, and incubated in 0.25% and 0.5% acetic anhydride for 15 min each.

The slides were incubated 1 hour at 56°C in a pre-hybridization buffer (50% formamide, 5X SSC, 0.1% Tween 20, 1 mg/ml yeast RNA, 10 mM DTT, 0.1 mg/ml heparin), then were incubated overnight at 56°C in the hybridization buffer (10% dextran sulfate in pre-hybridization buffer) plus the probe at a concentration of 0.5 ng/μl., then washed four times with washing buffer (50% formamide, 5X SSC, 0.1% Tween 20) for 15 min at 56°C and four times for 10 min at RT with MAB buffer (99.4 mM maleic acid, 167 mM NaCl, 0.1% Triton x-100, pH 7.5).

Samples were incubated with blocking buffer (MAB buffer, 1% BSA and sheep serum) for 30 min at RT, incubated overnight at 4°C with anti-digoxigenin-AP antibody (Roche, Basel, Switzerland) diluted 1/500 in blocking buffer, washed with MAB buffer 6 times for 15 min, then incubated in TMN for 5 min, and finally placed in NBT/BCIP developing solution (Roche, Basel, Switzerland). Slides were examined with a light microscope (Primo Star, Zeiss, Oberkochen, Germany) and photographed with a calibrated camera (AxioCam ICc1, Zeiss, Oberkochen, Germany) using AxioVision software (version 4.6, Zeiss, Oberkochen, Germany).

### Generation of cDNA and qPCR

qPCR was performed using 12 cysts tissues (six vesicular and six racemose samples). Samples of vesicular larvae were divided into the bladder wall and scolex using a scalpel to isolate the total RNA of each structure separately. The samples were homogenized in 1ml of TRIzol reagent (Invitrogen, Carlsbad, CA) for standard RNA isolation, concentrations were determined using a UV spectrophotometer (Nanodrop Products, Wilmington, DE). cDNA was generated from 500 ng of total RNA using the High-Capacity cDNA Reverse Transcription Kit with MultiScribe RT polymerase and random primers (Applied Biosystems, Foster City, CA) in a final volume of 20 μl per reaction and, incubation for 10 min at 25°C followed by 60 min at 37°C, 5 min at 95°C on a SimpliAmp Thermal Cycler (Applied Biosystems, Foster City, CA). Real-time PCR (qPCR) was performed in 10 μl reaction volumes using SsoAdvanced Universal SYBR Gene Supermix (BioRad Laboratories, Hercules, CA) with designed primers for *plk1* (TsM_001148400) (Forward: 5’-TCGACAATCTTGCCCGTAATC-3’) (Reverse: 5’-GGTGTAGTCTTTATTCGCCTCTG-3’) and *gapdh* genes (TsM_000056400) (Forward: 5’-TCCAAGAGATGAATGCCAATGC-3’) (Reverse: 5’-CAGAAGGAGCCGAGATGATGA-3’). qPCR reactions, run in triplicate, used the following cycling parameters: preincubation of 2 min at 50°C and 10 min at 95°C followed by 40 cycles of 15 sec at 95°C and 1 min at 60°C, on a Lightcycler 96 System (Roche, Basel, Switzerland). The scolex was used as a calibration sample and we expressed the results as relative to the expression of the *gapdh* gene using the 2^-ΔΔCT^ formula [27].

### Immunofluorescence

The slides were submerged in 10 mM citrate buffer (10 mM citric acid, 0.05% Tween 20, pH 6.0) for 30 min at 95°C, incubated for 30 min with a blocking solution (PBS pH 7.2, 0.05% Tween 20, 0.1% Triton X-100, 2% goat serum, 2% BSA) in a humid chamber at RT, then incubated overnight at 4°C with rabbit anti-phospho-histone H3 (Ser10) antibody (Cell Signaling Technology, Danvers, MA) diluted 1/800 in PBS, washed three times for 2 min with washing solution (PBS pH 7.2, 0.05% Tween 20) and then incubated for 30 min at RT with the fluorescein-labeling goat anti-rabbit antibody (Jackson ImmunoResearch Lab, West Grove, PA) diluted 1/500 in PBS. The sections were then washed with PBS and mounted with VectaShield mounting medium with DAPI (Vector, Laboratories, Burlingame, Ca). The human cell line U251 was included as a proliferation control. Images were captured by confocal microscopy (Zeiss, LSM880, Oberkochen, Germany).

### Cell isolation and culture conditions

The sections of racemose larvae selected for cellular isolation were washed in 50 ml of PBS for 5 min at room temperature and processed according to Spiliotis [28] with modifications. Eight volumes of a solution of 0.025% trypsin and 0.01% EDTA were added, incubated for 15 min at 37°C, and then the tube was shaken very gently, turning it slowly over and back for approximately 3 min. The supernatant was collected in a separate tube and 35 ml of PBS was added to the tube containing the samples and shaken vigorously for 3 min. The supernatant was collected again and 35 ml of PBS were added again. The samples were shaken vigorously until they became transparent and floated to the top of the tube; the supernatant was once again collected. The three collected fractions were pooled and then centrifuged for 10 min at 400G, the supernatant was discarded and the pellet was resuspended with RPMI 1640 medium (supplemented with 10% of heat-inactivated fetal bovine serum, 1mM sodium pyruvate, 100 U/ml penicillin, 100 g/ml streptomycin and 0. 25 g/ml amphotericin B, 0.01 mM nonessential amino acids, 0.2 mM L-glutamine, 1.6 μM β-mercaptoethanol, 25 mM HEPES; all from Gibco-Invitrogen, Gaithersburg, MD) and the cells were distributed in 12-well plates (1.5 ml of cell suspension per well) and cultivated at 37°C and 5% CO_2_. The plates were observed daily with an inverted microscope.

### Evaluation of supernatants by Ag-ELISA

Cell isolated from racemose larvae and *T*. *crassiceps* cysts were resuspended and seeded in 12-well plates at three density conditions: low density (< 100 cells per well), medium density (< 1000 cells per well) and high density (> 100000 cells per well), incubated for 48 hours at 37°C and 5% CO_2_ and the supernatants were collected. Briefly, 100 μl/well of mAb (TsW8) at 2 μg/ml was used to coat 96 well flat-bottomed plates (Nunc MaxiSorp) using carbonate-bicarbonate buffer pH 9.6 (0.05 M NaHCO_3_/Na_2_CO_3_, Sigma, St. Louis, CA) and left for 30 min at 37°C. Then, 100 μl/well of blocking solution (PBS pH 7. 4, 0.05% Tween-20 and 1% non-fat milk), was added and incubated for 30 min at 37°C and washed 5 times using PBS pH 7.4, 0.05% Tween-20. 100 μL of supernatant, pretreated with 5% trichloroacetic acid was added (in duplicate), and incubated for 30 min at 37°C with gentle shaking. After 5 washes, 100 μL of the second MAb (TsW5-biotinilated at 2 μg/ml diluted in blocking solution) was added and incubated for 30 min at 37°C on a shaker. After another 5 washes, 100 μL of streptavidin diluted 1/10 000 in blocking solution was added, and incubated for 30 min at 37°C with gentle shaking and washed again 5 times. Finally, 100 μL of *o*-phenylenediamine (OPD/H_2_O_2_) diluted in citrate buffer was added as substrate/chromogen, and incubated in the dark for 15 min. The reaction was stopped with 50μL of 2N H_2_SO_4_. Plates were read at 650 nm using a VersaMax ELISA microplate reader (Molecular Devices), and each result was expressed as the OD ratio. E/S antigens were prepared following the previously published protocol [29] and employed as positive controls, using 50 vesicular cysts of *T*. *solium* and racemose larvae.

### RT-PCR assay

For the detection of host cell contamination, specific primers for the human *β-actin* gene (NM_001101.5) were designed. Total RNA of three primary cell cultures of racemose larvae were isolated using TRIzol reagent (Invitrogen, Carlsbad, CA), and concentrations were determinated using a Nanodrop (Nanodrop Products, Wilmington, DE). cDNA was generated from 500 ng of total RNA using the High-Capacity cDNA Reverse Transcription Kit (Applied Biosystems, Foster City, CA) in 20 μl per reaction and, incubation for 10 min at 25°C followed by 60 min at 37°C, 5 min at 95°C on a SimpliAmp Thermal Cycler (Applied Biosystems, Foster City, CA). PCR was performed with specific primers (Forward: 5’- AGCCATGTACGTTGCTATCC-3’) (Reverse: 5’- CGTAGCACAGCTTCTCCTTAAT-3’) with a protocol of 30 cycles of 45 sec at 95°C, 45 sec at 57 C, and 45 sec at 72 C. PCR reactions were subsequently separated on a 1% agarose gel, ethidium bromide was added to the gel before electrophoresis to a final concentration of 0.5 μg/ml, followed by separation at 100V for 1 hour and visualized under UV light. cDNA generated from total human blood cells was employed as a positive control.

### Growth curves and insulin effect

Cell growth was measured to determine the doubling time of germinative cells and to evaluate the *in vitro* action of insulin. The cells of three primary cultures were resuspended and an aliquot of each cell suspension was diluted for cell counting and viability evaluation (by trypan blue dye exclusion) using a hemocytometer. We seeded 10,000 cells isolated from *T*. *solium* racemose larvae and *T*.*crassiceps* cysts in 24-well plates. Considering 70 pmol/L as the baseline (the normal level of insulin in blood during fasting), three concentrations of insulin (35, 70, and 140 pmol/L) were evaluated in triplicate and the cell count performed every 24 hours. The doubling time was obtained by applying the following formula (using logarithm to base 10):

$$\mathrm{Doubling}\;\mathrm{time}=\frac{\mathrm{Duration}\;*\mathrm{Log}\;(2)}{\mathrm{Log}\;(\mathrm{final}\;\mathrm{concentration})‐\mathrm{Log}\;(\mathrm{initial}\;\mathrm{concentration})}$$

For *in situ* hybridization and immunofluorescence using isolated cells, we proceeded with the protocols used for tissue samples, with modifications. The samples were fixed in PFA-PBS overnight at 4°C and all steps were performed in Eppendorf tubes and centrifuged between each step.

### Statistical analysis

Non-parametric statistic, Mann-Whitney U test for two groups, were calculated using Prism software (Graphpad, San Diego, CA) for comparisons of the gene expression between scolex (vesicular larvae) and bladder wall (racemose larvae) or bladder wall (vesicular larvae) and bladder wall (racemose larvae). Differences with *p* values <0.05 were considered statistically significant. The growth curves are presented as a dot plot with the mean ± standard deviation. Quantitative comparisons of insulin effect in cell proliferation *in vitro* were analyzed by one-way ANOVA and *p* values <0.05 were considered statistically significant.

## Results

**Fig 1:**
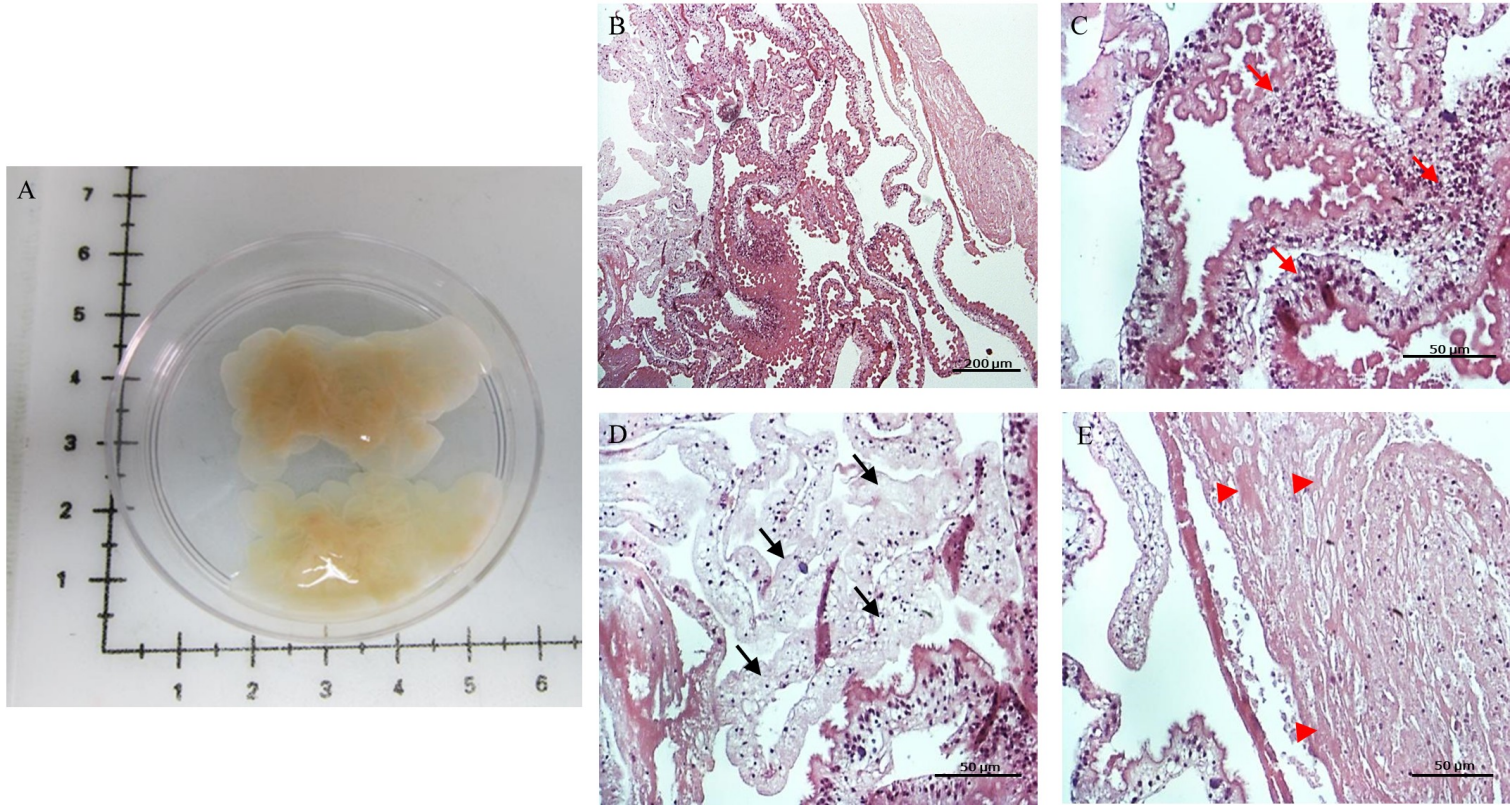
Racemose larvae sample. Macroscopic view of representative sample (A) and HE stain of the sample (B). Three selected regions with different histological characteristics; region with intact or viable tissue (C), the tegument has normal morphology with microtriches on the surface (red arrows are pointing to hypertrophic/integrity regions); region with tissue degeneration (black arrows) (D); region with a high degree of necrosis (E) characterized by the absence of nuclei (arrow head).

We were able to obtain and process six racemose larvae samples collected over 12-months. Randomly selected sections of all racemose samples revealed variable histopathology features (Fig 1B) including hypertrophic regions or regions with tissue integrity (Fig 1C); other parts of the sample showed tissue degeneration (Fig 1D) or necrosis (Fig 1E). The proportion of these regions varied in each sample and many times these regions were neighbors to each other. Macroscopic identification of these regions is difficult to perform and required the use of vital stains to ensure the selection of viable tissue samples for analyses.

**Fig 2:**
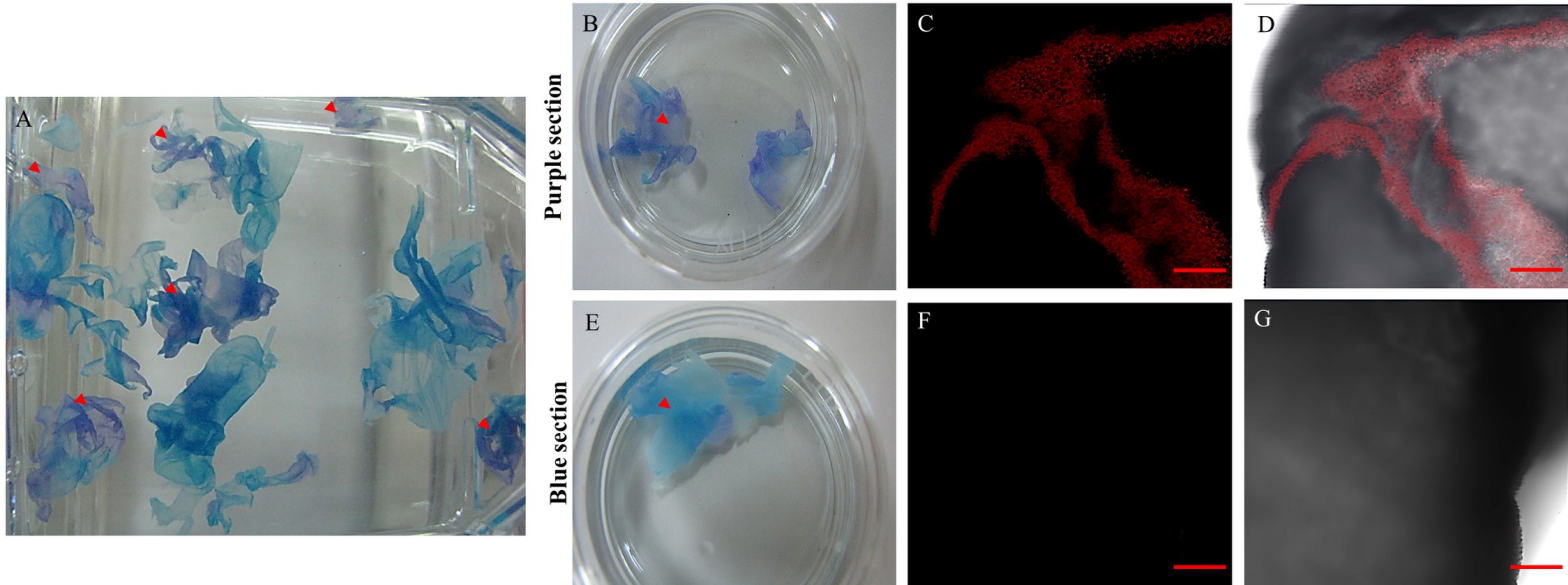
Racemose larvae sample after double staining. Macroscopic view of the sample (A), purple (arrowheads) and blue sections were placed in separate Petri dishes for confocal microscopy. The purple sections and blue sections in petri dishes (B,E) and 20X confocal pictures were taken, red for mitotracker (C,F) and merge with brightfield (D,G). In all the purple sections a positive signal for the mitotracker was observed. After 48 hours, the staining was repeated and the same results were obtained. Scale bar: 200 μm.

Viability was assessed using mitochondrial activity as the main indicator. After the double staining with MitoTracker and methylene blue, the tissue of racemose larvae appeared either blue or purple (Fig 2A). Under confocal microscopy; the purple color is caused by the combined color of methylene blue and Mitotracker indicating functioning mitochondria (Fig 2B–2D). Therefore, purple sections were selected for further study.

**Fig 3:**
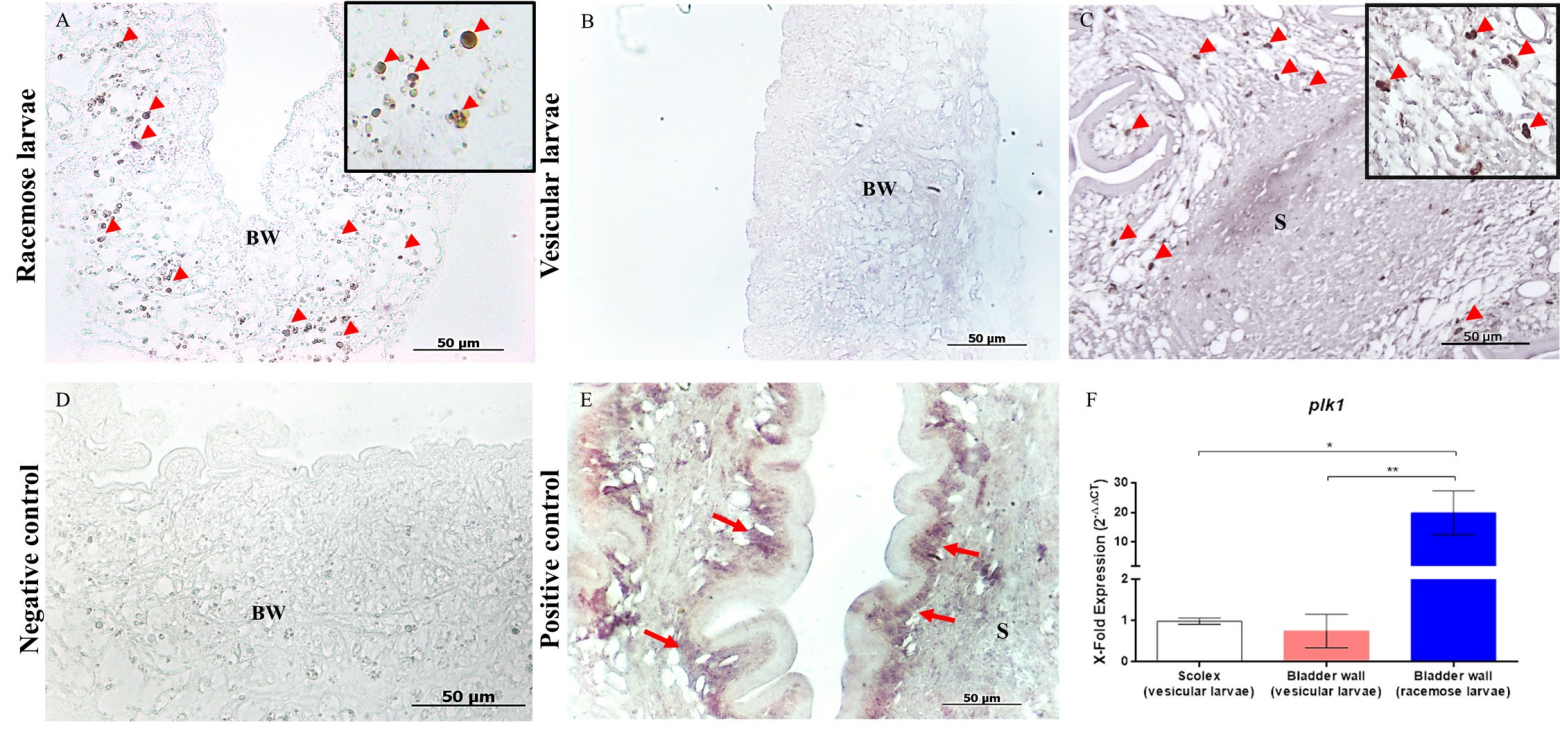
In situ identification of proliferative cells in *Taenia solium* cyst samples by ISH. *Plk1*-expressing cells were observed in racemose larvae (A) and were limited to the neck of scolex in vesicular larvae (C) (arrowheads). Proliferative cell were absent in the bladder wall of vesicular larvae samples (B). A higher magnification image is inserted (A,C). A sense probe of *plk1* (D) was used as a negative control and antisense probe for actin as a positive control (E) in vesicular larvae where strong staining of muscle fibers was observed (arrows). Differential expression of *plk1* gene in racemose form (F). Quantitative PCR of cDNA from 500 ng of total RNA from racemose form compared to the scolex and bladder wall of vesicular form. Results are normalized to the housekeeping *gapdh* gene and expressed as fold increase in *plk1* expression. Statistically significant differences in levels of gene expression are indicated by asterisks (Mann-Whitney U test). Asterisks represent level of significance: *: p<0.05; **: p<0.01. BW: Bladder wall; S: Scolex.

The presence and localization of proliferating cells were determined in the cysts of *T*. *solium* (vesicular larvae obtained from infected pigs and racemose larvae obtained from subarachnoid NCC patients) and *T*. *crassiceps*. In racemose larvae, *plk1*-expressing cells were present in the bladder wall in all samples of the racemose larvae (Fig 3A) and noticeably absent in the bladder wall in all samples of the vesicular form (Fig 3B). Additionally, we evaluated the expression level of *plk1*, and as shown (Fig 3F), there is a significant increase in *plk1* expression in racemose larvae compared to the scolex and bladder wall of vesicular form.

**Fig 4:**
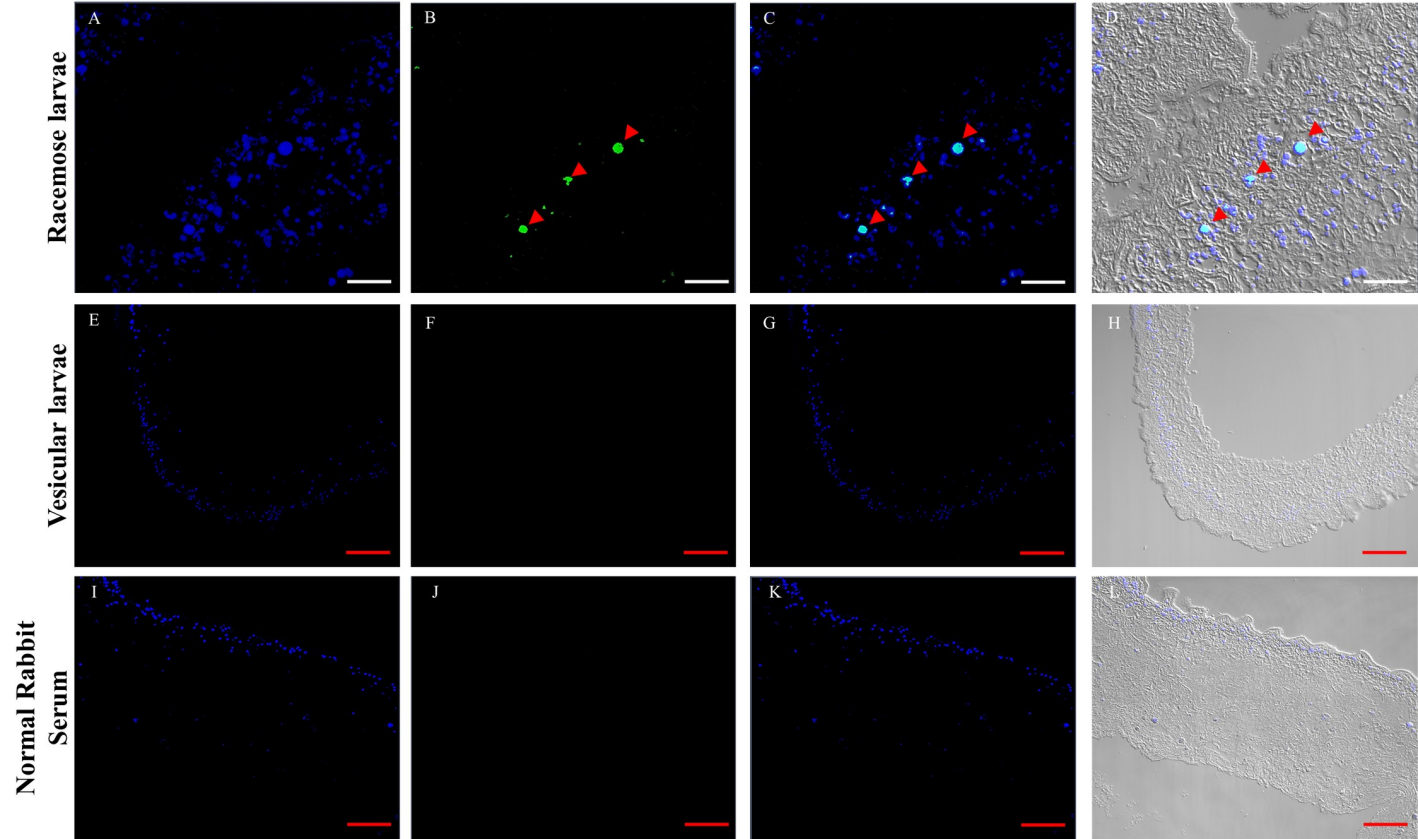
In situ identification of mitotic cells in *Taenia solium* cyst samples by IF. IF assays performed on racemose and vesicular larvae. The nuclei were stained with DAPI (A,E,I), positive cells to phospho histone H3 (arrowheads) were observed only in racemose larvae (B), merge (C,G,K), merge + brightfield (D,H,L). Sample of racemose larvae was incubated with normal rabbit serum and used as a control. Images captured with a 20X objective; racemose larvae: zoom 3, vesicular larvae and control: zoom 1.White scale bar: 20 μm; red scale bar: 50 μm.

As a complementary assay, we analyzed the mitotic activity by immunofluorescence to detect histone H3 phosphorylated in serine 10 (Fig 4). The location of the hybridization stain and the immunofluorescence signal indicates the presence of mitotically active cells only in the bladder wall of racemose larvae.

**Fig 5:**
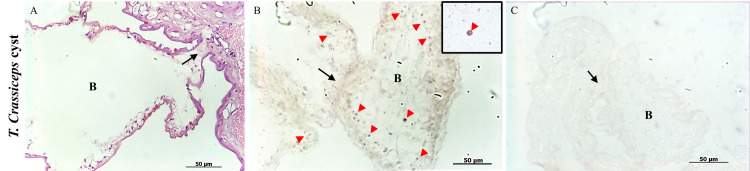
In situ identification of *plk1*-expressing cells in samples of *Taenia crassiceps cyst*. HE staining (A) and ISH on *Taenia crassiceps* cyst (B) were also performed; positive cells were observed in the region of the "buds" formation (black arrows) and along the bladder wall of these structures (arrowheads). A higher magnification image is inserted. A sense probe (C) was used as a negative control. B: Bud.

The ORF strain of *T*. *crassiceps* cysts lack scolices, are much smaller than *T*. *solium* vesicular cyst, and normally propagate by asexual budding, which is a process whereby multiple buds develop from a mother cyst to form new cysts. Cells expressing *plk1* were present over the entire vesicular bladder wall but were concentrated at growth regions of developing buds (Fig 5B), which is consistent with their essential role in cyst multiplication.

**Fig 6:**
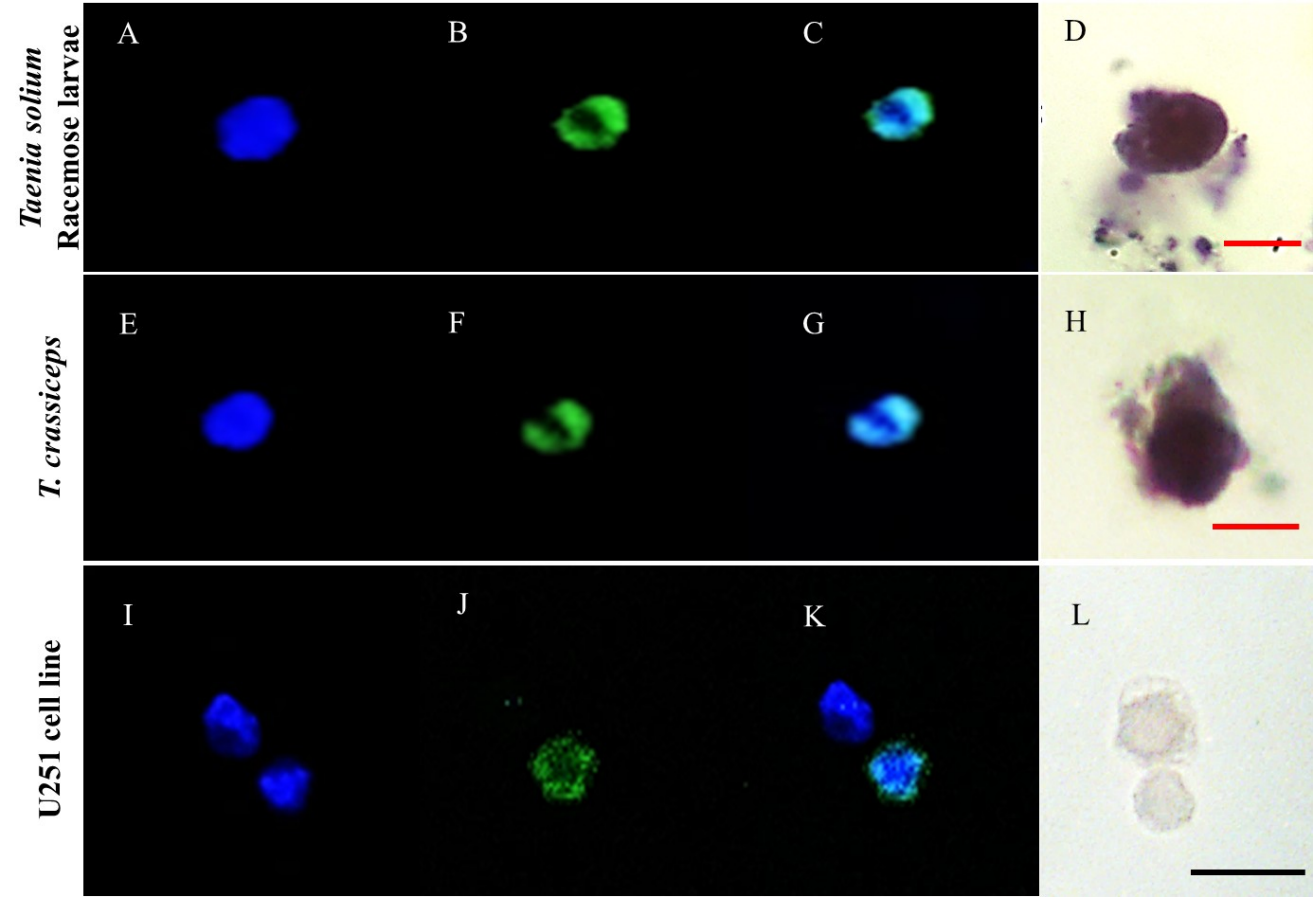
Proliferative cells isolated from *Taenia solium* racemose larvae and *Taenia crassiceps* cyst. Culture of cells isolated from racemose larvae and from *T*.*crassiceps* cyst by enzymatic digestion. The nuclei were stained with DAPI (A,E,I), and for the presence of phospho histone H3 (B,F,J), the merge of both signals (C,G,K) was observed with confocal microscope. Separate cells (D,H) were tested for *plk1*transcripts (*in situ*). The human glioblastoma cell line U251 was used as a control for proliferation (J,K) and specificity (L). Red scale bar: 10 μm; Black scale bar: 20 μm.

**Fig 7:**
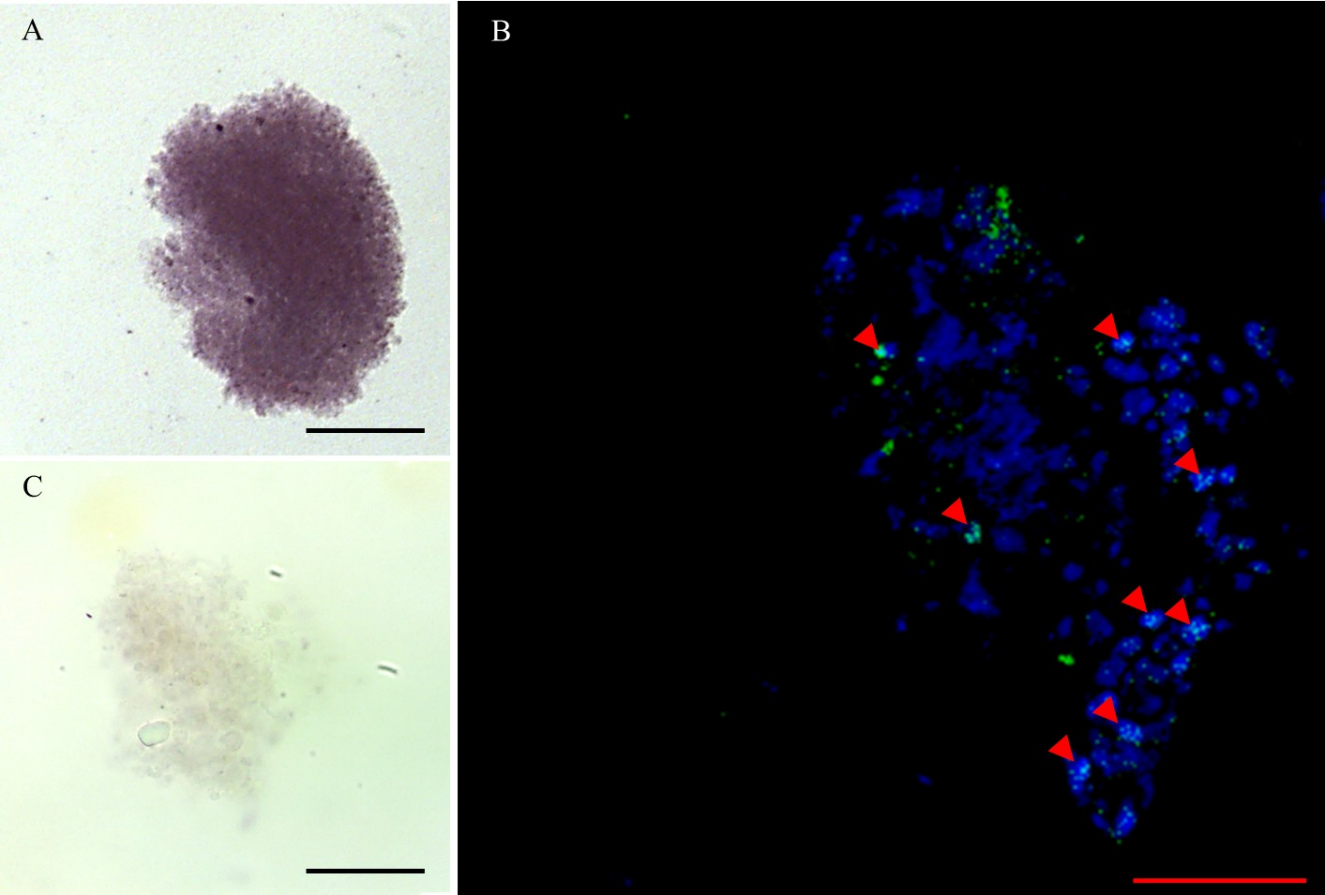
IF in proliferative cell clusters from racemose larvae. The clusters express *plk1* (A) and were reactive to phospho histone H3 (arrowheads) (B) and the nuclei were stain with DAPI. A sense probe (C) was used as a negative control. Scale bar: 50 μm.

Cell isolation released cells from the bladder wall of racemose larvae with diverse morphological characteristics. After the first week of culture in the presence of reducing agents, only proliferative cells survived, this was confirmed by viability assays in cell cultures (S2 Fig). These increased in number and spread all over the bottom of the cell culture plate, but were easily dislodged from the well surface. They showed morphological characteristics similar to those described in the neoblasts of free-living planarians [30] with a prominent nucleus, little cytoplasm, and a diameter of 8–12 um approximately (Fig 6). After the third week of culture, *plk1*-expressing cells formed aggregates (Fig 7) that increased in size. It was made up of different sized cells along with extracellular material. (S3 Fig). Cultured cells grew for at least 6 months (15 passages) with low saturation density.

**Fig 8:**
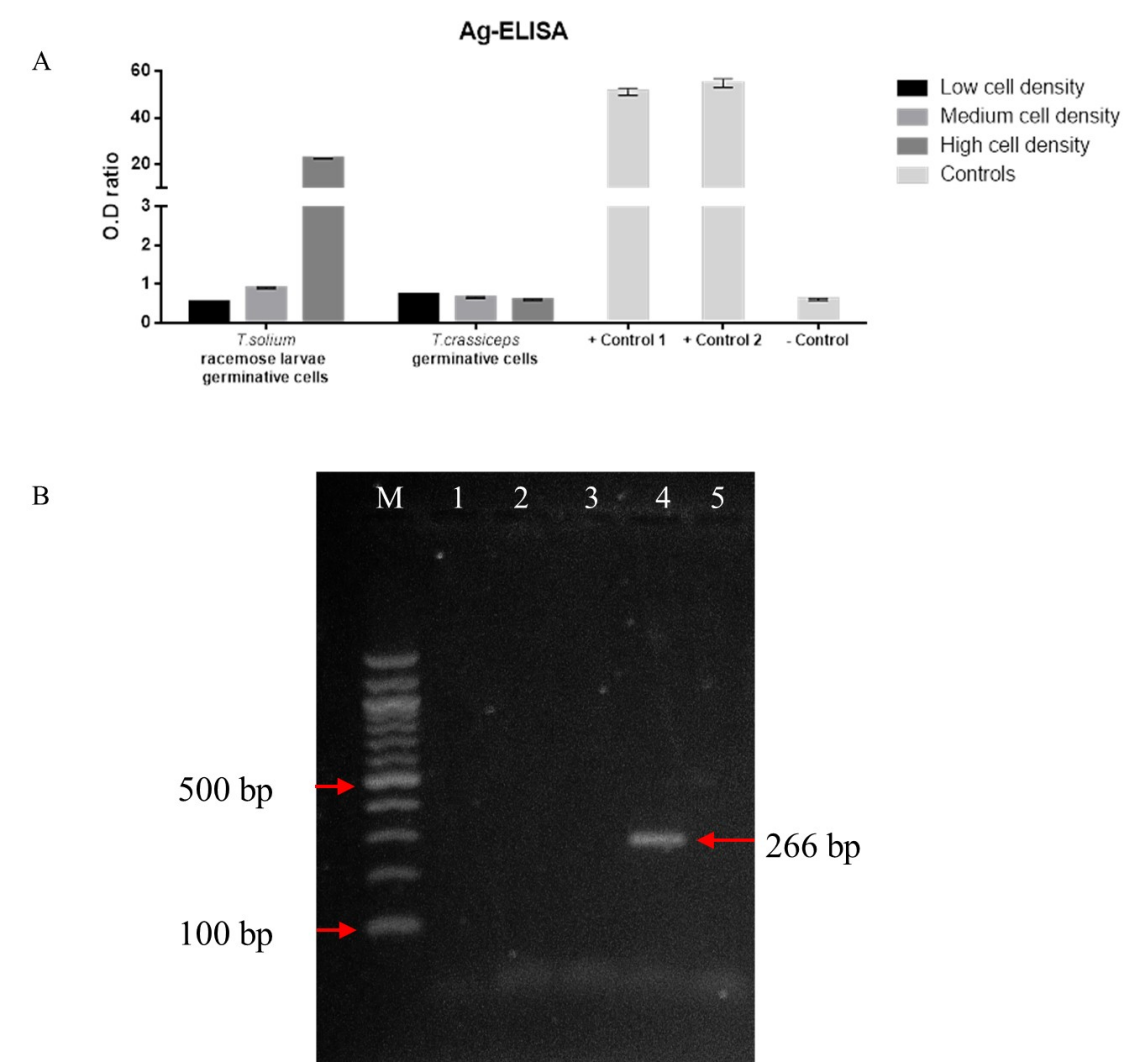
Evaluation of supernatants by Ag-ELISA and RT-PCR of cell cultures. Evaluation of supernatants obtained from proliferative cell cultures with different densities using anti-*T*. *solium* monoclonal antibodies (A). The values were expressed as a ratio by dividing the optical density (OD) of the samples by the OD of the cut-off value. An ELISA ratio >1 was considered as positive. Excretory/Secretory antigens were used as positive controls (1 and 2) and supplemented RPMI culture medium as the negative control. Agarose gel electrophoresis of RT-PCR (B). cDNA was generated from the total RNA of three cell cultures of racemose larvae. The specific amplification product (~266 bp) for *β-actin* gene was only observed in the positive control human blood cell cDNA (left arrow). M: 100 bp DNA ladder; 1–3: samples of proliferative cell cultures; 4: positive control; 5: master mix.

To confirming the parasitic origin of the cells supernatants from our different cultures were tested for *T*. *solium* antigen using a monoclonal-based in-house ELISA assay [31]. Only samples with a high density of racemose cells produced significant antigens levels, these results are also consistent with the usual high levels of antigens detected for cases of subarachnoid NCC [31]. Supernatants from *T*. *crassiceps* cells tested negative (Fig 8A).

To rule out any possible contamination of host cells in racemose cell cultures, total RNA isolated from three primary cell cultures was evaluated by RT-PCR using specific primers for the human *β-actin* gene. After performing electrophoresis to visualize the amplification product, we observed that the parasite cell cultures are free of host cell contamination (Fig 8B).

**Fig 9:**
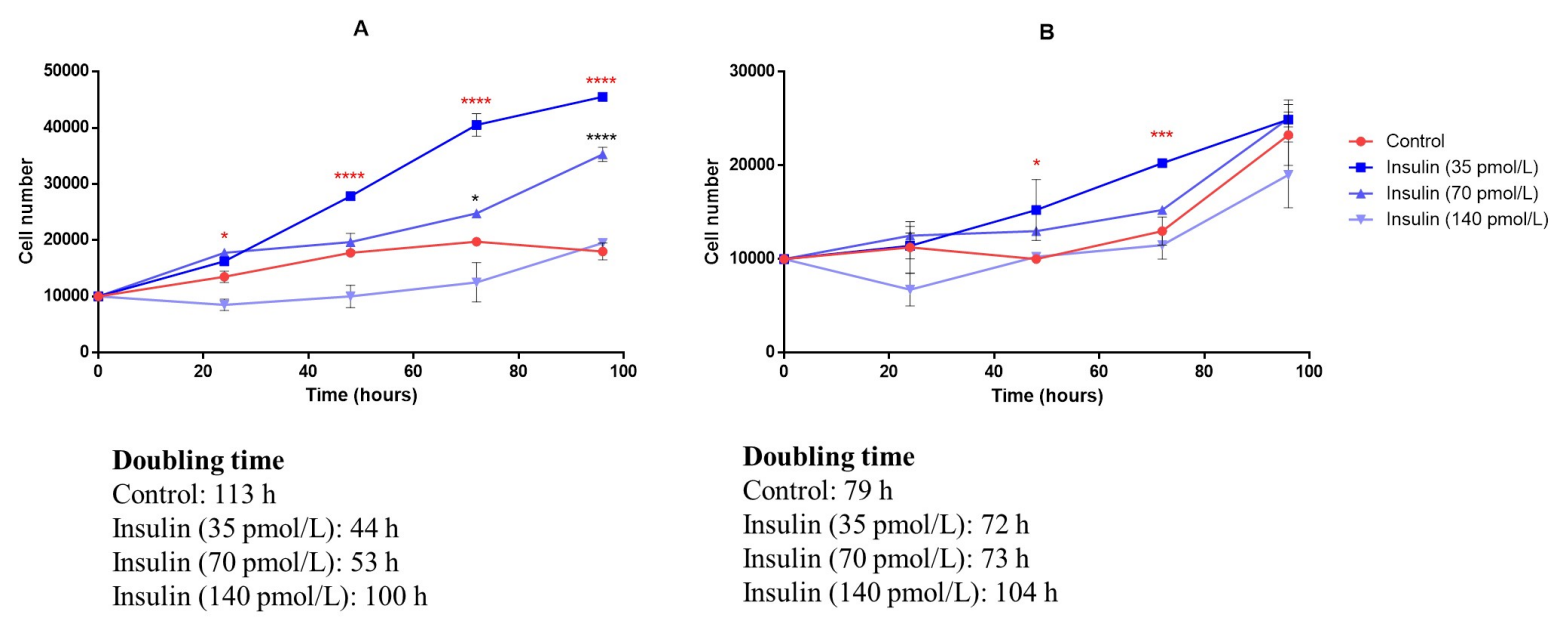
Growth curves of proliferative cells and insulin effect. Growth curves of cells isolated from *T*.*solium* racemose larvae (A) and *T*.*crassiceps* cyst (B). Insulin at 35 pmol/L produced a significant increase in racemose larvae cells after 24 h (red asterisks) and 70 pmol/L after 72 h with (black asterisks). In the case of *T*. *crassiceps*, cells increased significantly with 35 pmol/L after 48 hours (red asterisks). Higher concentration (140 pmol/L) slowed growth in both cultures. Asterisks indicate statistically significant differences between groups (treated and control). One-way ANOVA test (* *p* < 0.05; *** *p* < 0.001; **** *p* <0.0001).

**Fig 10:**
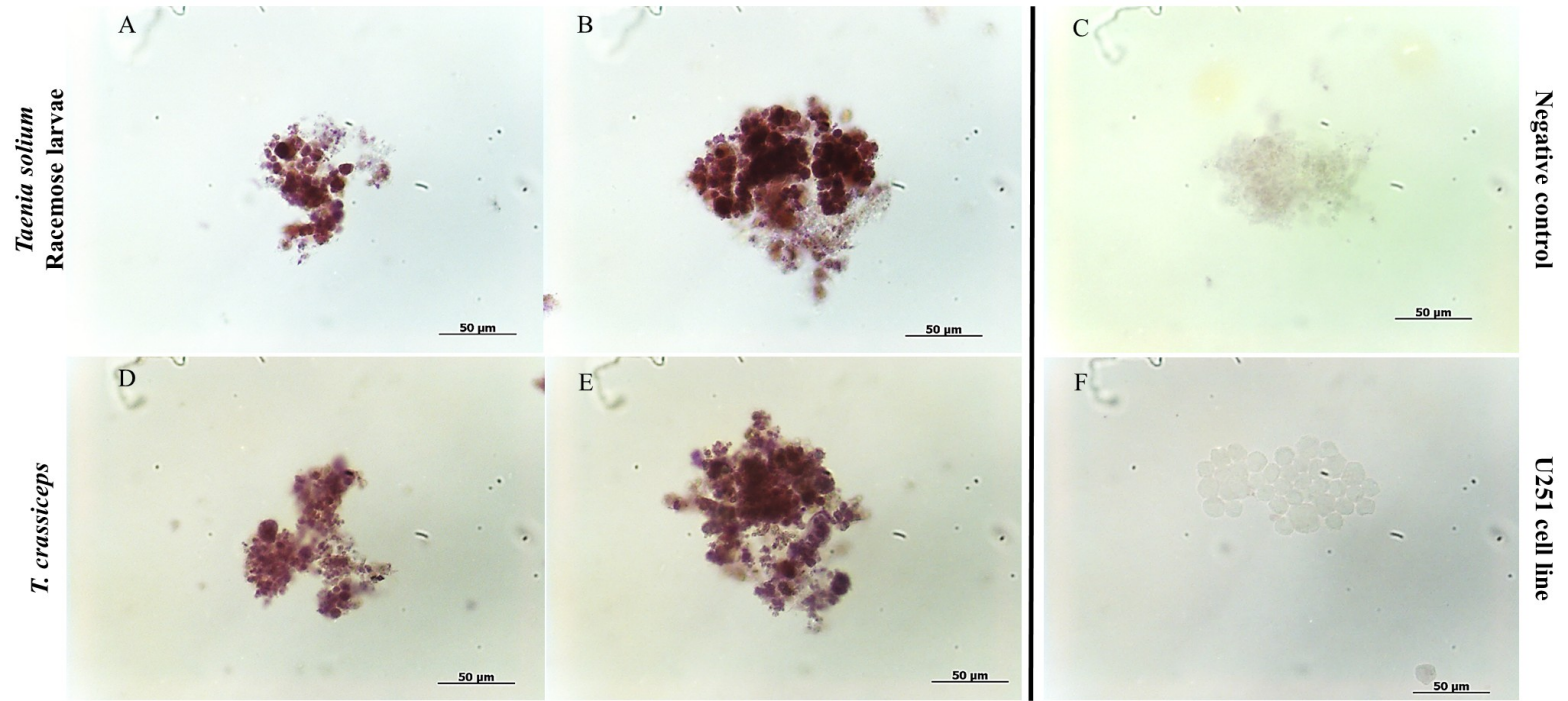
ISH assay in proliferative cells isolated from *T*.*solium* larvae and *T*.*crassiceps* cyst. The cells express *plk1* before (A,D) and after being cultured 1 week with insulin (B,E). A sense probe (C) was used as a negative control. The human glioblastoma cell line U251 (F) was used as a control for specificity.

The doubling times of cells isolated from racemose and *T*. *crassiceps* larval cysts were 113 h and 79 h, respectively. The stationary phase of the growth curve was not reached in racemose or *T*. *crassiceps* cell cultures perhaps because of slow growth and sparse density. The addition of insulin at 35 pmol/L produced a significant increase in the growth of racemose larvae cells after 24 hours and at 70 pmol/L after 72 hours (Fig 9A). *T*. *crassiceps* cell only showed a significant increase in growth with insulin at 35 pmol/L after 48 hours (Fig 9B). Additionally, insulin decreased doubling time in both cultures but racemose larvae cells showed enhanced growth at a lower insulin concentration compared to *T*. *crassiceps* indicating an increased sensitivity to insulin (Fig 9). Higher concentrations of insulin slowed growth in both cultures. We observed that the pro mitotic action of insulin did not alter *plk1* expression (Fig 10). These findings suggest the importance of hormones from host endocrine and paracrine systems in the development and growth of the parasite [32].

## Discussion

The nature and character of racemose NCC have been poorly studied. Zenker suggested that racemose cysts and normal vesicular cysts both originated from *T*. *solium* after he identified degenerated *T*. *solium* scolices within racemose form [33]. More recently their identity was confirmed by molecular methods [34]. Additionally, their rather constant association of racemose cysts with other forms of NCC in the same patient also suggested their origin related to *T*. *solium* [35]. By the 20^th^ century, there was a general acceptance that the massive involvement characteristic of racemose disease seen in autopsies was due to proliferation [36]. However, *in vivo* racemose cyst growth and extension has only recently been documented and even then only rarely by MRI imaging, which allows detailed characterization as well as serial observations over time in untreated or inadequately treated patients [9]. Direct evidence of proliferation and the processes involved are otherwise lacking.

Plk1 plays multiple roles in the cell cycle [37] and is dynamically regulated during mitosis at the level of its expression, activation, and distribution within the cell [38]. Previous studies have shown that *plk1* is highly expressed in most human cancer, and its over-expression is associated with poor prognosis in patients [39]. Here we observed a large number of *plk1*-expressing cells in the bladder wall of the racemose larvae and showed that these cells are absent in the bladder wall of the vesicular form (Fig 3A and 3B). One possible reason for the unexpectedly high number of *plk1* positive cells is a dysregulation in the gene expression, and the messenger would be present in the G1/S phase of the cell cycle, as has been reported in cancer cells [40,41]. Furthermore, the expression levels of *plk1* reveal significant differences between both forms of the parasite (Fig 3F).

As a complementary approach, we observed a lower number of phosphohistone-H3 positive cells (average: 96 cell per mm^2^ of tissue) compared to *plk1*-expressing cells (average: 695 cell per mm^2^ of tissue) (Fig 4); these results were consistent with the fast transition thought M phase of the cell cycle, as has been described in other cestodes [42,43]. These results are consistent with the uncontrolled tumor-like growth of the racemose bladder wall.

Our observations suggest that the proliferative cells present in the bladder wall of the racemose larvae are germinative cells because they present morphological characteristics and proliferative capacity similar to those described in other cestodes [17,20]. Further studies are needed to identify the totality of germinative cells and to characterize subpopulations according to the expression of specific cell markers.

Since proliferative cells are only present in the vesicular cyst scolex [23,44], their presence across the racemose bladder wall suggests that they result from the incorporation of the scolex tissue in the expanding bladder wall in early phases of racemose larval development. Racemose larvae grow continually indicating tumor-like parasite. Possible mechanisms inducing formation of racemose form include degeneration and loss of tissue organization perhaps caused by their location in the subarachnoid spaces, a part of the brain lacking the nutritional benefits of the brain parenchyma. This could lead to degeneration of the scolex, and loss of normal developmental coordination, including the inhibition of cell proliferation and organization of tissues.

Additionally, overexpression of genes that promote growth, loss of function through downregulation of growth repressing genes, as a consequence of mutations or epigenetic factors, or an unusual and abnormal response to host growth factors like insulin could contribute and explain the development of the racemose form. These are concepts that require exploration in future studies.

Racemose larvae have properties similar to tumors, which in itself suggest abnormal growth [10]. Besides the continued proliferation and growth over long periods, up to decades, their tissue morphology is also abnormal. Like tumors of higher animals, racemose larvae resemble the tissues from which they are derived, but their organization and structure are abnormal and varied [10,45]. Differences are found between racemose samples and within larvae. The ability of racemose larvae to proliferate has profound implications for treatment. In the example of parenchymal form, which has no potential to proliferate, effective treatment is relatively short and generally kills most cysts [46]. Shortly after treatment, a profound immune response is induced, which appears to be important and possibly essential to effect cyst killing [7]. In contrast, similar short courses of treatment are not curative for racemose disease; regrowth is frequent [7]. Therefore, curative treatment requires prolonged treatment. This is consistent with the requirement to kill all proliferating cells.

We established primary cell cultures of *T*. *solium* and *T*. *crassiceps*. Only proliferative cells survived in the presence of reducing agents (Fig 6), increased in number and showed enhanced growth in response to insulin (Fig 9). We did not observe a different effect of insulin as previously reported [32], perhaps because the racemose larvae of *T*. *solium* and the cyst of *T*.*crassiceps* proliferate from the bladder wall in a similar manner. These cultures do not result in complete cyst regeneration. We only observed the formation of clusters (S3 Fig); this perhaps due to differences in the culture medium that we used compares to *E*. *multilocularis* cultures [28].

The cross-reaction between antigens of *T*. *solium* and *T*. *crassiceps* allows the use of *T*. *crassiceps* in serodiagnostic assays for human cysticercosis [47]. However, our ELISA assays did not detect parasite antigens in supernatants obtained from *T*. *crassiceps* cell cultures (Fig 8A), perhaps due to the low sensitivity of the assays and different monoclonal antibodies; this is consistent with the wide range of sensitivity and specificity in ELISA assays based on different *T*. *solium* and *T*.*crassiceps* antigens [48].

The importance of these cultures resides in their potential to allow studies in developmental cell biology of *T*. *solium* and other cestodes, germinative cell biology, cell metabolism, and as a way to identify novel drug treatment. Compounds such as BI2536 [49] that inhibit *plk1* gene expression or metformin [50] that suppresses the development of parasite *E*. *multilocularis* can be tested in these cultures for their ability to kill or inhibit growth. Elimination or inhibition of germinative cells would likely control growth or kill racemose larvae and may limit parenchymal cyst survival and prevent recovery from small doses of damaging drugs.

## Supporting information

S1 Fig Schematic outline of the study. The processes from sample collection to the establishment of the primary culture (left) and rationale for each step (right). ISH: in situ hybridization; IF: immunofluorescence; FFPE: formalin-fixed paraffin-embedded.
(TIF)

S2 Fig Viability of proliferative cells and clusters. Cells isolated from racemose form were cultivated with mitotracker. 40X confocal images were taken in brightfield (A, E), DAPI (B, F), mitotracker red (C, G) and merge (D). White scale bar: 5 μm; red scale bar: 10 μm.
(TIF)

S3 Fig Enlarged picture of *pkl*1-expressing cell cluster from racemose larvae. The cells tend to form aggregates consisting of cells of different sizes (arrowheads) surrounded by extracellular material (arrows).
(TIF)
