# Supplementary figures and images for: Identification and culture of proliferative cells in abnormal Taenia solium larvae: Role in the development of racemose neurocysticercosis
Source: PLoS Negl Trop Dis. 2021 Mar 22;15(3):e0009303. doi: 10.1371/journal.pntd.0009303 (PMC8016263; doi:10.1371/journal.pntd.0009303)

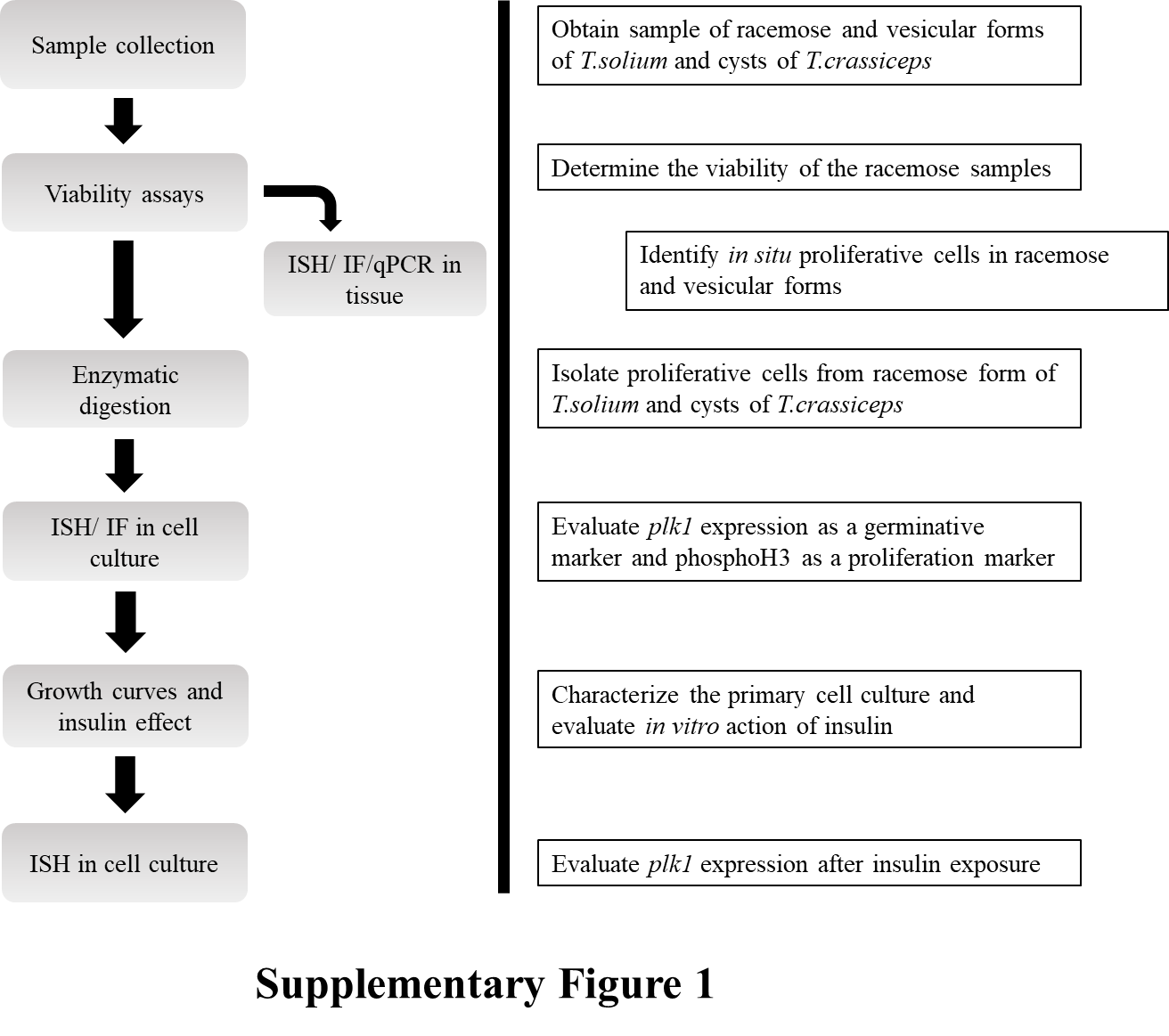

Supplement: S1 Fig — The processes from sample collection to the establishment of the primary culture (left) and rationale for each step (right). ISH: in situ hybridization; IF: immunofluorescence; FFPE: formalin-fixed paraffin-embedded. (TIF) [file pntd.0009303.s001.tif]

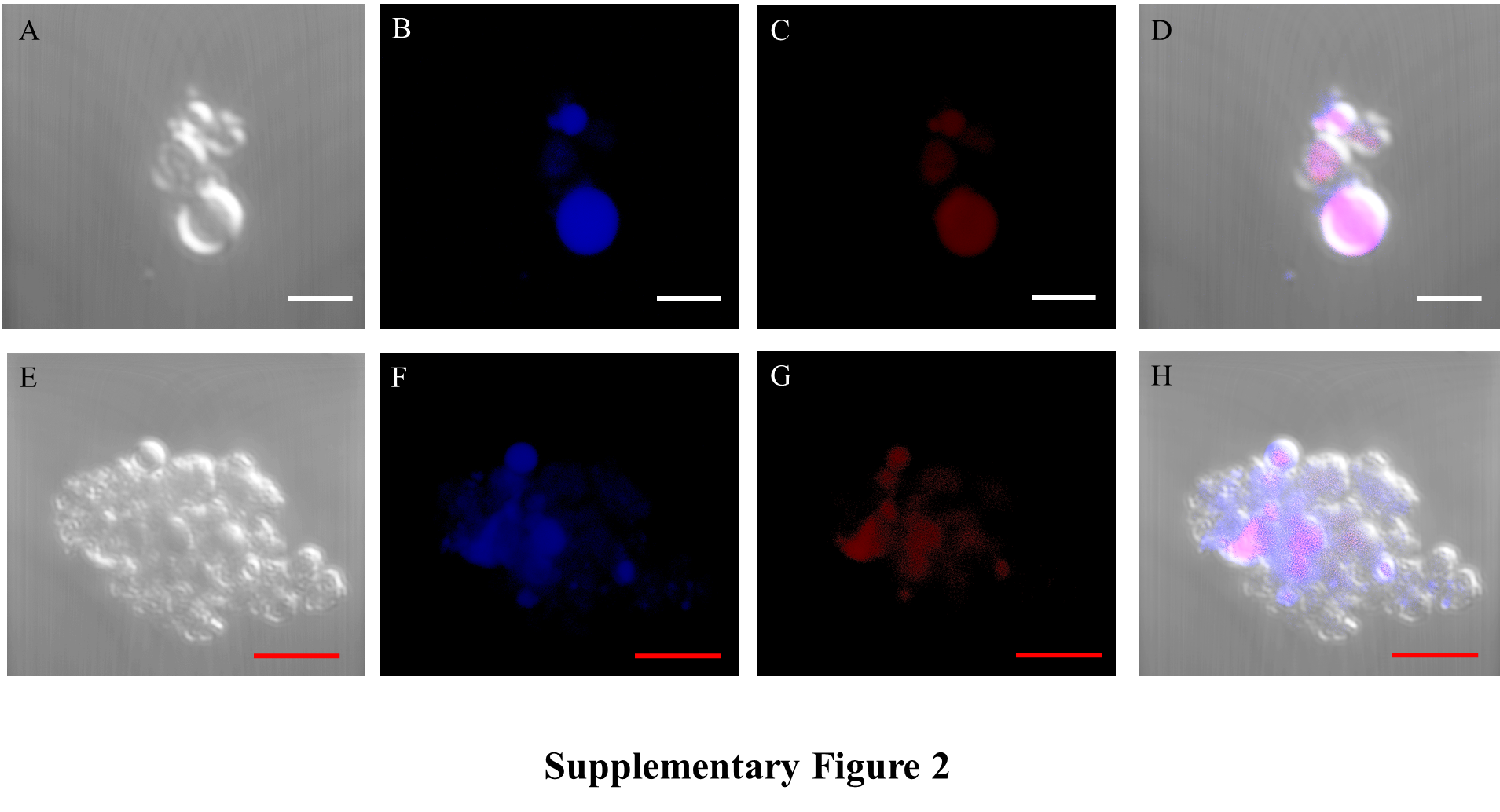

Supplement: S2 Fig — Cells isolated from racemose form were cultivated with mitotracker. 40X confocal images were taken in brightfield (A, E), DAPI (B, F), mitotracker red (C, G) and merge (D). White scale bar: 5 μm; red scale bar: 10 μm. (TIF) [file pntd.0009303.s002.tif]

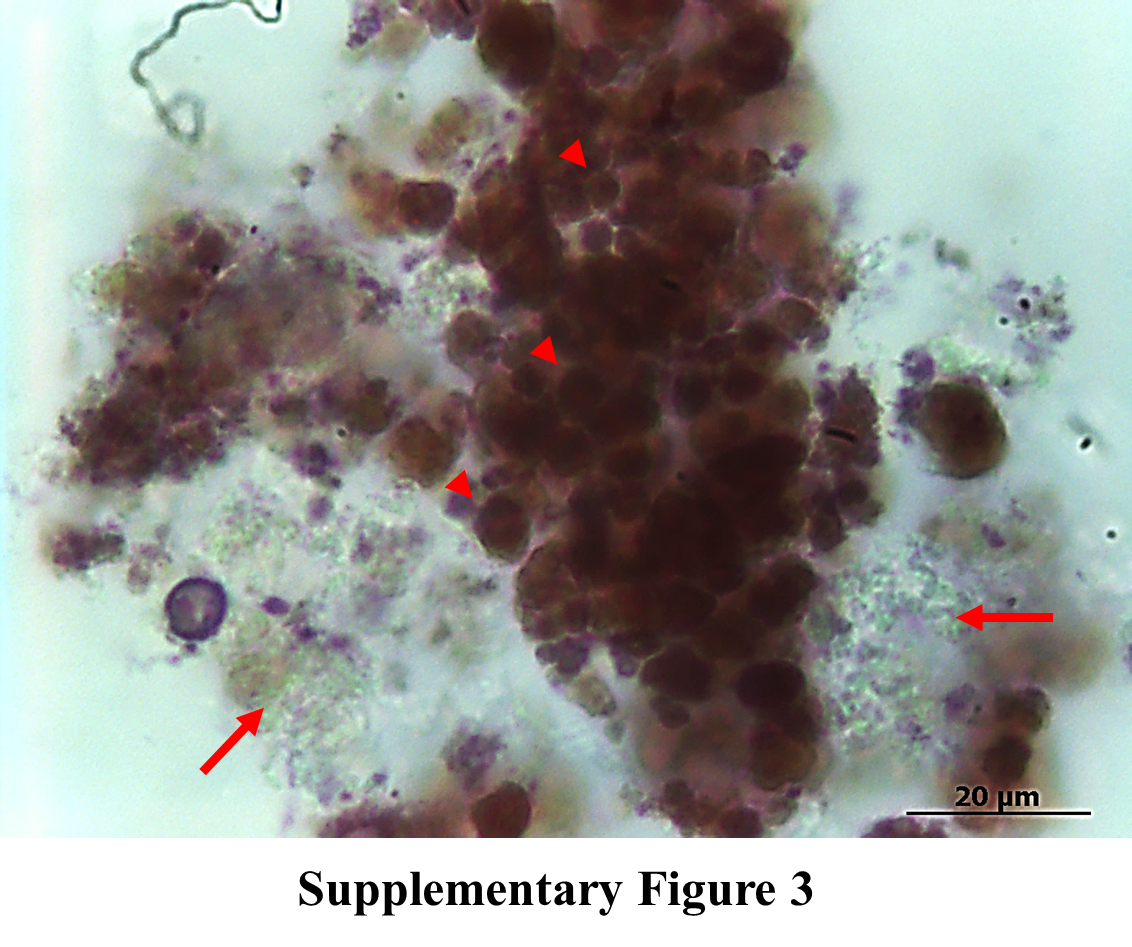

Supplement: S3 Fig — The cells tend to form aggregates consisting of cells of different sizes (arrowheads) surrounded by extracellular material (arrows). (TIF) [file pntd.0009303.s003.tif]
